# Measurement of Spectro-Temporal Processing by Cochlear Implant Users: Effects of Stimulus Level and Validation of an Online Implementation

**DOI:** 10.1097/AUD.0000000000001676

**Published:** 2025-06-06

**Authors:** Dakota Bysouth-Young, François Guérit, Lidea Shahidi, Robert P. Carlyon

**Affiliations:** 1 Cambridge Hearing Group, MRC Cognition and Brain Sciences Unit, University of Cambridge, Cambridge, UK.

**Keywords:** Cochlear implant, Psychophysics, Spectro-temporal ripple, Spectro-temporal, Speech perception

## Abstract

**Objectives:**

Evaluating adjustments to cochlear implant (CI) settings is challenging as recipients need time to adapt for optimal speech test performance. The Spectro-Temporal Ripple for Investigating Processor EffectivenesS (STRIPES) test, a language-independent measure of spectro-temporal resolution, has been validated with Advanced Bionics and Cochlear CI systems. This study investigates if performance on the STRIPES test varies with presentation level in a loudspeaker setup and its relationship with outcomes on the British Coordinate Response Measure (CRM) test. In addition, it extends the use of STRIPES and its online version “webSTRIPES” to Med-El CI systems.

**Design:**

A prospective, single-blind, two-session repeated-measures study was conducted with 10 CI users. The first session included three blocks: pre-test webSTRIPES, STRIPES at three loudspeaker presentation levels (50, 65, and 75 dB SPL), and post-test webSTRIPES. The second session measured the speech reception threshold (SRT70) for CRM sentences with a time-reversed speech masker, presented at the same three levels.

**Results:**

Presentation level did not significantly affect STRIPES ripple density thresholds or SRT70 for CRM sentences. A significant correlation was found between STRIPES loudspeaker and webSTRIPES thresholds. WebSTRIPES showed good-to-excellent test-retest reliability. The correlation between CRM SRT70 and STRIPES thresholds, while in the predicted direction, was not statistically significant, likely due to the small sample size (n = 7), which may have limited the power to detect a meaningful relationship.

**Conclusions:**

STRIPES and webSTRIPES ripple density threshold scores can be reliably measured with Med-El CI systems, unaffected by presentation level. The STRIPES test is a promising tool for assessing adult CI listener outcomes without requiring prolonged acclimatization to programming changes.

## INTRODUCTION

Cochlear implants (CI) improve speech perception in quiet and noisy listening situations compared with pre-implantation (Holden et al. 2013; Birman & Hassarati 2023). However, there is significant variability in outcomes (Boisvert et al. 2020) and despite extended acclimatization and/or auditory rehabilitation, even high-performing CI recipients still perform poorly on speech-in-noise tests and in ecologically valid listening environments when compared with normal-hearing listeners (Goldsworthy et al. 2013; Holden et al. 2016; Ma et al. 2023). Improving outcomes and reducing the significant variability in speech perception for CI recipients, especially in noise, has been the focus of considerable investigation given that open-set speech recognition is often regarded as the gold standard for assessing CI outcomes (Tamati et al. 2022). Efforts to enhance the performance of CIs in noisy environments have become a focal point of extensive research, spanning various approaches such as preprocessing noise removal paradigms, alternative stimulation methods, and the individualized customization of specific programming parameters (Carlyon & Goehring 2021). Using speech perception tests to acutely assess changes to CI settings poses a number of challenges including variability in test methodology and outcomes within a recipient (Brungart et al. 2022; Ma et al. 2023), and the need for adaptation to new settings to achieve optimal performance in speech tests (Davis et al. 2005). While providing a period of acclimatization before experimental assessment is an option, it risks subjecting participants to potentially inferior settings, and long-term acclimatization to a recipient’s routine clinical program may still bias results (Schvartz-Leyzac et al. 2017). To address this, spectral and spectro-temporal ripple tests have been developed that provide an efficient means of evaluating the effects of changes in CI settings, offering valuable insights into improvements in auditory perception without the issues of speech-based assessments (Won et al. 2007; Azadpour & McKay 2012; Aronoff & Landsberger 2013; Archer-Boyd et al. 2018).

Spectro-temporal modulations are integral to complex acoustic signals like speech, and effective speech understanding relies, in part, on an individual’s ability to detect and discriminate these modulations. Ideally, such tests should be very sensitive to the amount of speech-relevant information being delivered at the auditory nerve: while one would not expect a perfect correlation with speech-based assessments (because of factors such as vocabulary and working memory), they should be very sensitive to distortion of speech content, for example, the effect of spectral blurring (Goehring et al. 2020), or the difference between two site-selection speech processing strategies (Goehring et al. 2019).

**Fig. 1.**
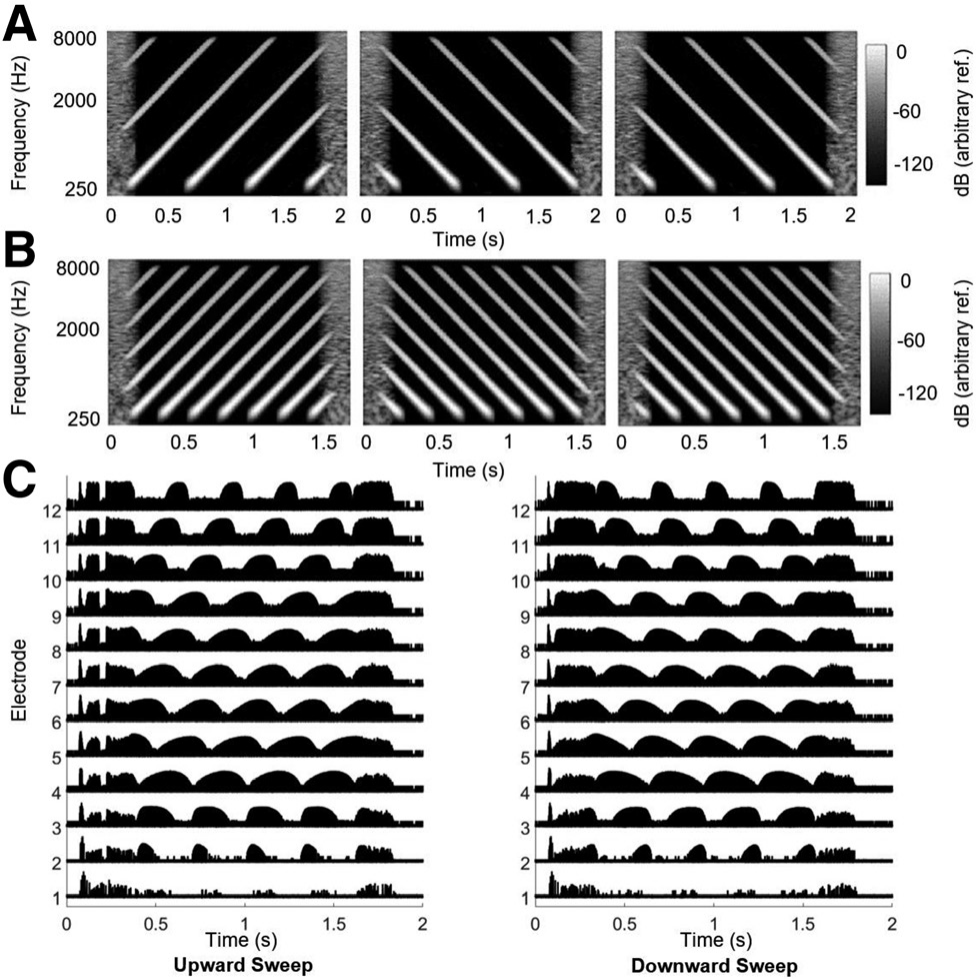
Spectrograms and electrodograms of STRIPES stimuli. A, Schematic representation of the spectrograms of the three stimuli present in one trial of the STRIPES test. In this example, the signal, consisting of upward sweeps, is present in interval one, with a ripple density of 2.5. B, STRIPES stimuli with a ripple density of 5. C, Electrodogram plots with a density of 3, using a MED-El Combi 40+ implant detector box and Sonnet 2 sound processor with FS4 processing strategy streamed via an Audiolink. An upward sweep (left) and downward sweep (right). The *Y* axis shows the electrode number (1 Apical – 12 Basal) and the *X* axis represents time in seconds.

The Spectro-Temporal Ripple for Investigating Processor EffectivenesS (STRIPES) test is an adaptive, nonspeech test initially developed with CIs manufactured by Advanced Bionics (AB) (Archer-Boyd et al. 2018). The STRIPES test involves spectro-temporal ripple discrimination, where listeners are presented with concurrent sinusoidal sweeps (with a roved onset frequency) that increases or decreases in frequency over time, requiring listeners to discriminate between downward- and upward-sweeping stimuli (Fig. 1A). Increasing the temporal overlap, or ripple density (RD), between each sweep, increases the difficulty such that a higher density score indicates better performance (Fig. 1B). The amplitude modulation (AM) rate and depth for a given electrode do not differ systematically between upward and downward sweeps. The roving of the onset phase and inclusion of noise at the start and end of each stimulus help minimize the potential influence of onset and offset cues. Archer-Boyd et al. (2018) initially validated the STRIPES test using vocoder simulations with normal-hearing listeners and CI listeners using direct audio input. Inspection of electrodograms before testing revealed the absence of within-channel cues, demonstrating that spectro-temporal processing (involving comparisons using both frequency and time) was necessary to perform the task. This distinction is important given another commonly used test, the spectral-temporally modulated ripple test (SMRT) (Aronoff & Landsberger 2013), has been shown to be confounded by within-channel AMs that listeners can exploit to complete the task (Zhou et al. 2020). The STRIPES test has been demonstrated to be reliable across test sessions with routine clinical devices and programming strategies in free-field testing (Archer-Boyd et al. 2020) as well as an online implementation termed “webSTRIPES” (Archer-Boyd et al. 2023). STRIPES has been shown to correlate with speech-in-noise and SMRT tests at a fixed level (van Groesen et al. 2023b), and, importantly, to predict which of two experimental fitting algorithms produces the best speech-in-noise condition for individual listeners (Goehring et al. 2019). Although the test shows promise as a research and clinical tool for investigating listeners’ spectro-temporal resolution, uncertainties remain regarding the influence of presentation level and the test’s relevance to devices beyond the AB and Cochlear CI systems.

The primary aim of this study was to assess whether performance on the STRIPES test is influenced by presentation level. Previous studies with CI listeners have used either the most comfortable listening level or 65 dB SPL, while normal-hearing listeners using vocoded stimuli have been tested at 70 dB SPL. The impact, if any, of varying presentation levels on performance in CI listeners remains unclear. This is of interest for two reasons; first, webSTRIPES requires participants to adjust the presentation level to a comfortably loud sensation level, so it is important to know the extent to which the test is robust to differences in level across sessions and between participants. Previous research has shown that multichannel modulation detection thresholds (MDTs) outperform single-channel MDTs when loudness differences are not compensated, as CI users utilize envelope information across channels instead of focusing on the best-performing one (Galvin et al. 2014). Moreover, spectro-temporal sensitivity in CI users cannot be predicted by spectral or temporal modulation detection alone (Zheng et al. 2017). Given the degradation of spectral cues in CI users, the interaction between temporal and spectral dimensions may enhance spectro-temporal detection, highlighting the need for further research on their interplay at different presentation levels. Second, if STRIPES thresholds are affected by presentation level, an additional validation of the test would be provided by the finding that a similar variation occurred for speech perception. This would avoid the effects of between-listener differences in linguistic and cognitive skills that would be expected to reduce (but not eliminate) simple correlations between the overall level of performance on STRIPES and speech tasks. Such an evaluation is provided here by measuring the speech reception threshold (SRT) in competing noise for the British Coordinate Response Measure (CRM) test with the same participants and presentation levels as used for STRIPES. Previous measures of the effects of level on speech and nonspeech tests and their interaction have produced mixed results, with the variation being possibly due to differences in the range of levels, device type, and (de-) activation of automatic gain control (AGC), as well as choice of tests between studies (Won et al. 2007, 2011; Azadpour & McKay 2012; Boyle et al. 2013; Holden et al. 2016; Brochier et al. 2017; Goehring et al. 2019; van Groesen et al. 2023b). This study extends previous research by comparing a spectro-temporal test with speech-in-noise tests at various presentation levels. It is anticipated that STRIPES will correlate with CRM and that if there is an effect of level, it will be in the same direction for the two tasks.

A secondary aim was to extend the corpus of data validating STRIPES and webSTRIPES from the AB and Cochlear devices tested previously (Archer-Boyd et al. 2018, 2020, 2023) to recipients of Med-El CI systems (Innsbruk, Austria). A potential issue with the test’s use with the Med-El device arises from concerns raised by Winn and O’Brien (2022) regarding the use of spectral ripple tests to measure spectro-temporal resolution in CI listeners. Winn and O’Brien argued that the spectral density transmitted by a CI is inherently limited by the number of frequency channels and the bandwidth of each channel. If the spectrum is under-sampled, it leads to spectral aliasing, which can introduce distortions by adding new spectral components that were not present in the original signal. The density of variations in the frequency spectrum, indicating the complexity of the spectral pattern within an octave, can be defined in terms of ripples per octave (RPO). For a rippled spectrum with a certain number of RPO (N ripples), at least double that number of frequency channels per octave is required to properly represent the spectral envelope. Calculation of this limit depends on the bandwidths of the analysis filters, which are broader for the Med-El device due to the smaller number of stimulating electrodes (a maximum of 12 electrodes), resulting in a comparatively smaller upper RPO limit (1.1 RPO) compared with AB (2 RPO) or Cochlear (2 to 2.5 RPO) devices. While STRIPES and webSTRIPES have been shown to be robust in previous studies, with only 1 participant (AB24) exceeding the critical limit for spectral aliasing with a Cochlear or AB CI system (Archer-Boyd et al. 2023), the grouped average thresholds for both paradigms have been near the upper RPO limit for Med-El devices. This difference in RPO limits suggests that the Med-El system could be more prone to spectral aliasing. Furthermore, the fine structure processing (FSP) sound coding strategy has an extended low-frequency allocation that shifts the passband of the lowest-frequency channel below the stimulus spectral composition of STRIPES. Given STRIPES thresholds have been shown to increase monotonically from 4 to 16 channels in normal-hearing participants using vocoded stimuli (Archer-Boyd et al. 2018), reducing the available number of channels for task completion could result in lower (poorer) thresholds or increased variability for participants using Med-El devices.

Another difference between Med-El’s FSP sound coding strategy compared with that implemented in other devices is that it attempts to enhance the representation of temporal fine structure in auditory signals. The FSP sound coding strategy does this by encoding each positive-going zero crossing of a filtered waveform with brief bursts of pulses (typically 5000 to 10,000 pulses per second) in up to the four lowest-frequency channels (e1 to e4), while the rest of the channels employ a high-pulse-rate continuous interleaved sampling (CIS) strategy. Studies comparing Med-El CIS only to FSP strategies have been mixed with some showing a significant benefit in speech perception with FSP (Vermeire et al. 2010; Kleine Punte et al. 2014) and others showing no significant benefit (Müller et al. 2012; Dillon et al. 2016). Comparisons of the number of FSP channels have also failed to show a significant group-level benefit (Riss et al. 2014; Müller et al. 2020). CI listeners may be unable to use the additional cues from the FSP strategy in a consistent manner, potentially due to the limitation of temporal processing by CI listeners who have poor discrimination of rate changes at rates higher than approximately 300 pps (Kong et al. 2009), which is lower than the highest zero-crossing rate conveyed by the FSP strategy. In addition, fine structure is not always in phase across all frequency channels and these pulse bursts might produce extraneous or misleading cues, for example, a complex or unclear pitch (Carlyon & Goehring 2021; de Groote et al. 2025). Despite the sparse spectral density of the STRIPES stimuli, these spurious cues could affect task performance, with some listeners either ignoring or attempting to use this information. If this occurs, we would expect to see highly variable thresholds or outliers in the results for participants using either STRIPES paradigm.

To extend the clinical use of STRIPES and webSTRIPES tests to Med-El users, it is clearly important to assess the effect of presentation level and reliability of performance with the Med-El device. This evaluation will help determine whether STRIPES can be applied in studies exploring novel programming methods or processing strategies with Med-El devices. We hypothesize that the results will remain below the spectral aliasing threshold, exhibiting consistent variability across different paradigms and trials. As an additional comparison, we provide the mean data from previously published webSTRIPES outcomes in context with our findings.

## MATERIALS AND METHODS

### Study Design

A prospective two-session repeated-measures study design was used. The initial testing session consisted of three blocks: pre-test webSTRIPES, STRIPES at three loudspeaker presentation levels (50, 65, and 75 dB SPL), and post-test webSTRIPES. WebSTRIPES testing was performed at a single comfortable presentation level and tested twice so as to account for any acute learning or order effects, as well as to enable an estimate of test-retest reliability. The final session consisted of a modified version of the CRM speech test (Semeraro et al. 2017) presented via loudspeaker at three presentation levels (50, 65, and 75 dB SPL). Loudspeaker presentation levels in both sessions were counterbalanced and randomly assigned. Participants were not informed at the start of each test what sound level was to be presented.

### Participants

Demographic and device information are shown in Table 1. Ten Med-El listeners took part. All used an FS4 sound coding strategy as their routine clinical strategy. As an additional measure, we also tested three Cochlear CI users using the Advanced Combination Encoder strategy. A laboratory-owned Sonnet 2 (Med-El) or Nucleus 7 (Cochlear) sound processor was used for all testing, after loading the participant’s routine clinical map settings. For all streaming of webSTRIPES stimuli, a laboratory-owned AudioLink (Med-El) or minimic2+ (Cochlear) was used with mixing ratios between the microphone and streaming devices set to “streaming only” to minimize any extraneous noise. Ethical approval was obtained from the National Research Ethics Committee for the East of England. Before commencing the experiments, listeners gave their informed consent to participate and were informed that they could withdraw from the study at any point. Listeners were paid for taking part and travel expenses were reimbursed.

**TABLE 1.** Participant demographic details including participant ID, sex, age in years, duration implanted in years, etiology of hearing loss and if acquired prelingually or postlingually, sound processor used, implant and array type, clinical strategy, pulse width, stimulation rate, Maplaw/compression ratio, as well as the individual number of any deactivated electrodes

Participant ID	Sex	Age (yrs)	Duration Implanted (yrs)	Etiology Acquired Pre/Post	Sound Processor	Implant/Array	Clinical Strategy	Pulse Width (μsec)	Stimulation Rate (pps)	Maplaw/Compression Ratio	Deactivated Electrode
MED01_01	F	43	10	Unknown/postlingual	Sonnet 2	Concerto/Flex 28	FS4	25.42–35.42	1277	500/3:1	None
MED02_02	M	78	13	Measles/postlingual	Sonnet 2	Sonata/Flexsoft	FS4	60.42	806	500/3:1	12
MED03_03	M	78	11	Unknown/postlingual	Sonnet 2	Concerto/Flex 28	FS4	13.75–27.08	750	500/3:1	None
MED04_04	F	45	11	Unknown/postlingual	Sonnet 2	Concerto/Flex 28	FS4-p	13.75–27.08	806	500/3:1	12
MED05_05	F	65	11	Unknown/postlingual	Sonnet 2	Concerto/Flex 28	FS4	18.75–32.50	806	500/3:1	12
MED07_07	M	75	10	Measles/postlingual	Sonnet 2	Concerto/Flex28	FS4	18.75–27.92	1296	500/3:1	12
MED08_08	F	72	10	Progressive/postlingual	Sonnet 2	Concerto/Flex 28	FS4	18.75–27.92	1215	500/3:1	None
MED09_11	M	74	10	Unknown/postlingual	Sonnet 2	Concerto/Flex 28	FS4	20.42–30.42	1357	500/3:1	12
MED10_12	M	33	5	Unknown/postlingual	Sonnet 2	Synchrony 2/Flex 28	FS4	21.25–33.75	1261	500/3:1	None
MED11_13	F	76	12	Unknown/postlingual	Sonnet 2	Concerto/Flex28	FS4	18.75–23.33	806	500/3:1	1
C47_06	M	79	0.5	Otosclerosis/postlingual	Nucleus 7	CI612	ACE	37	900	-	None
C39_09	M	67	1	Unknown/postlingual	Nucleus 7	CI622	ACE	37	900	-	None
C35_10	F	76	2.5	Infections/postlingual	Nucleus 7	CI622	ACE	50	900	-	22

### STRIPES

The implementation of the STRIPES test procedure was identical to that reported by Archer-Boyd et al. (2020) with two exceptions (total number of reversals and the inclusion of loudness judgments), and is described briefly here. The test employs an adaptive 2-up/1-down procedure to measure the RD threshold, which determines the point at which the listener can no longer distinguish a target stimulus from two reference stimuli.

Stimuli are WAV files created by combining 1 sec-long exponential sine sweeps. These sweeps are presented concurrently and concatenated together, with each sweep moving up or down in frequency between 250 and 8000 Hz at a rate of 5 octaves per second. The starting phase of each sweep is randomized, with the starting frequency continuously roved from trial to trial. Sweep density is defined as the number of concurrent sweeps presented at one time. Non-integer density values are possible; for example, a density of 5.5 would mean that for 50% of the time, five swept sinusoids were present simultaneously (overlapped) and that for the other 50% of the time, six swept sinusoids overlapped. The delay between the start of each sweep is 1 sec divided by the density, such that at a density of 5, the delay between the start of each sweep is 200 msec. Given that the bandwidth of the stimulus spans 5 octaves, the number of RPO is equal to the density divided by 5 and is reported in units of RD. For instance, if RD = 1.1, then RPO = 0.22. As individual sweeps are 1 sec in duration, the AM frequency in Hz corresponds to the density. The equation for a single cycle of a STRIPES stimulus is provided in Archer-Boyd et al. (2020). The number of cycles, and hence the duration of the stimuli, decreases with increasing density, such that at least 2 unbroken single sweeps are presented in each stimulus regardless of the starting phase and that an integer number of cycles at each density is always presented. As a result, the duration of the STRIPES stimuli ranged from 2.07 sec (RD = 1.1) to approximately 1.3 sec at the highest densities. Each STRIPES stimulus includes at least two continuous, complete sweeps, regardless of starting phase, and an integer number of cycles at each density is always presented to ensure the start and end instantaneous frequencies of each stimulus are aligned, minimizing this potentially salient and confusing cue. To minimize the salience of onset or offset cues, each interval is masked by a 250 msec noise burst at both the beginning and the end.

The listener’s task is to identify the target interval, which features an upward sweep. This interval can be either the first or the last, while the other two intervals contain downward sweeps (Fig. 1A). The density of concurrent frequency sweeps is varied to adjust the difficulty, starting at a relatively easy RD of 1.1 (close to 1 peak per 5-octave sweep) and becoming progressively more difficult as the density increases. The adaptive 2-up/1-down procedure starts with an RD of 1.1 and adjusts the density per trial with an additive density step size of 0.5 RD (for the first four reversals) and 0.2 RD (for the last four reversals). The inter-stimulus interval was 0.6 sec of silence between noise bursts. The test concludes after 8 reversals to streamline the procedure in alignment with Archer-Boyd et al. (2023), as opposed to the 12 reversals used by Archer-Boyd et al. (2020). The final threshold of the run was calculated as the average of the last four reversals. Before testing, STRIPES stimuli were presented at 1.1 RD at the three presentation levels (50, 65, and 75 dB SPL). Listeners rated loudness on an 11-point scale (0 to 10, with 0 indicating no audible sound and 10 indicating an uncomfortably loud sound). The loudness ranking at each level was confirmed to ensure audibility and comfort, with a minimum rating of 5 (“comfortable but soft”) required at 65 dB SPL. For those with residual hearing, earplugs were used.

### webSTRIPES

The webSTRIPES procedure was identical to that used in Archer-Boyd et al. (2023) and its differences with the loudspeaker version are highlighted here. The webSTRIPES stimuli are drawn from a library of premade stimuli with 10 fixed starting-frequency rove values spread equally over one cycle, rather than the continuous rove used previously. The noise bursts are also included in the premade stimuli, rather than being independently generated for each trial. The stimuli are 96 kbps mp3 files instead of the lossless WAV files used in the loudspeaker version. Finally, the inter-stimulus interval was 1 sec, compared with the 0.6 sec used previously. WebSTRIPES is implemented in Just Another Tool For Online Studies (JATOS), an open-source, cross-platform web application for hosting online studies written in JAVASCRIPT (Lange et al. 2015). The test was hosted on a local server at the University of Cambridge and the participants completed the testing using a wifi-connected Dell Laptop computer in a sound-treated room connected to the streaming device. At the beginning of the test, participants were instructed to set the output level on the computer to a comfortable loudness using a STRIPES stimulus with an RD = 5. An example test can be run at the following url: https://lsr-studies-02.mrc-cbu.cam.ac.uk/publix/zgUz5wMrg2a (note that the task should initially be very easy for a normal-hearing person listening acoustically).

Training in both webSTRIPES and STRIPES involves presenting participants with a run of trials at the lowest RD (1.1), and with the correct answer being highlighted at the end of each trial. A score of greater than 4 out of 5 trials correct leads to progression to the main experiment, while a score of less than 4 trials correct results in presentation of the pretest screening again until the participant scored 4 or more trials correct. All participants passed the pretest screening and proceeded to the main task. For the main task, participants completed two runs, with a third run conducted if the RD threshold difference between the two runs exceeded 1 RD. The mean score from all runs per condition was used as the final score for analysis. After completing two or three runs at a presentation level for STRIPES, the level was adjusted to the next fixed level as determined by counterbalancing. This procedure, including training, was repeated until all presentation levels were tested, resulting in six to nine thresholds per listener. The total duration of the first session was between 2 and 3 hr including breaks and paperwork.

### CRM Test

A modification of the British corpus CRM test (Semeraro et al. 2017) was used to estimate the SRT (SRT70), equal to the signal to noise ratio (SNR) at which participants achieve 70.7% accuracy, at the three presentation levels. The closed-set target sentences consisted of 32 CRM sentences featuring the call sign “Charlie” followed by “go to *color number* now,” where *color* was one of four options (green, blue, red, or white) and *number* was one of eight options (the numbers 1 to 8). The target sentences were spoken by the same male target speaker and fixed in level throughout each trial. The SRT70 measurement utilized an adaptive 1-up 2-down method, considering trials correct only when both key words (color and number) were identified accurately. The masker on each trial was selected at random from a single female speaker in the same corpus (256 CRM sentences), which was time-reversed to minimize lexical impact while ensuring sufficient masking (Deeks & Carlyon 2004; Goehring et al. 2020). Both target and masker stimuli underwent root mean square equalization to −30 dB Full Scale. The masker utterance preceded the target by 500 msec and looped to persist throughout the target signal.

To familiarize participants, a practice task comprising five target sentences without a masker preceded formal test runs at each presentation level. A score of 4 or 5 correct led to the main experiment, while a score less than 4 resulted in presentation of the pretest screening again until the participant scored 4 or more correct. Only subject MED02 required a repeat of the practice task. For each run, the initial SNR was set at +20 dB, with the masker level varied in steps that started at 8 dB and halved after the first and third turns. The SRT70 estimate represented the mean SNR of the final eight reversals or all total reversals if fewer than eight occurred. Runs with fewer than six reversals were excluded. The final SRT70 score at each presentation level was calculated based on the average of two runs, or three runs if the first two were greater than 4 dB different and had more than six reversals. Session length was no longer than 1 hr for all participants.

### Technical Setup

All loudspeaker testing took place in a double-walled Industrial Acoustics Company (IAC Acoustics, Winchester, UK) sound-proof booth measuring 2 × 2 × 2 m^3^. A Dell Inspiron laptop (Dell Inc., Round Rock, TX) was used to present the stimuli through an RME Fireface UCX (RME, Haimhausen, Germany) external soundcard connected to a Genelec 8030C loudspeaker (Genelec Oy, Iisalmi, Finland). The loudspeaker was placed 0.75 m from the wall opposite the door, and 0.35 m from the wall adjacent to the door, at a height of 1.2 m to the center of the loudspeaker. The loudspeaker was deliberately placed off-axis in the room, to minimize the effect of room modes. An office chair was placed in front of the speaker, such that the listener’s ear was approximately 1 m from the loudspeaker at an orientation of 0°. Participants were instructed to minimize head movement relative to the loudspeaker. Levels were calibrated using a sound-level meter positioned 1 m from the loudspeaker at a height of 1.2 m, using a noise with the same long-term spectrum and root mean square level as the STRIPES or CRM stimuli. As a final check for STRIPES, the levels of upward and downward sweeping STRIPES at an RD of 10 were also measured at the same position. CRM testing was limited to a maximal combined output of 94 dB SPL which represented a −16 dB SNR and was more than 10 dB SPL lower than previous studies with a similar closed-set speech-in-noise test (Chen et al. 2023). All free-field testing was completed using custom GUIs with MATLAB software (MathWorks 2019).

### Electrodogram Recordings

Before conducting any participant testing, we assessed the STRIPES stimulus to identify any potential bias-inducing cues within the electrodogram. To achieve this, we programmed the laboratory’s Sonnet 2 processor, with a Med-El test implant box (COMBI 40+), and connected it to a Salae Logic Pro 16 multichannel analyser running Logic 2.4.10 software (Saleae Logic Pro 16 2022). This allowed us to sample all 12 channels with 12-bit resolution at a rate of 1.5625 MHz. To ensure consistency, we set maximum stimulation levels (*M* levels) uniformly across all electrodes while maintaining Threshold stimulation levels (*T* levels) at 8% of the dynamic range. We then programmed the same electrode map using the FS4 processing strategy with the test conditions: default adaptive intelligence and AGC enabled. Adaptive intelligence is Med-El’s environmental scene classifier which controls the noise cancelation and microphone directionality. In the sound-proofed setup, we positioned a KEMAR dummy in the same position as our participants, with the Sonnet 2 processor placed on the left ear.

We presented STRIPES stimuli to a single audio channel, while a separate channel was used as a trigger, ensuring precise time synchronization for subsequent analysis. Individual STRIPES stimuli were presented in both upward and downward sweeping directions, with varying ripple densities. These stimuli were presented at each designated presentation level and program using the loudspeaker configuration, as well as via streaming using the audiolink.

## RESULTS

### Electrodogram Recordings

The electrodogram depicted in Figure 1C illustrates upward (left) and downward (right) sweeps at an RD of 3 while streaming using the FS4 strategy and, as was the case for participant testing, with all processor features including the AGC turned on.

Electrode 1, under the FS4 strategy, defaults to a center frequency of 120 Hz and a bandwidth of 100 Hz, resulting in no stimulation on the most-apical electrode. Although not shown here, shifting the default “High Definition” CIS strategy (which does not produce the pulse bursts) to a matching FS4 frequency allocation table produced a similar response.

The sweep direction is evident from the differences in across-channel timing. For example, in an upward sweep, each envelope peak in channel 10 is immediately followed by a peak in channel 11. Conversely, in a downward sweep, each envelope peak in channel 10 is followed by a peak in channel 9. An additional potential cue arises from the envelope asymmetries on all electrodes, with the most basal and apical electrodes having a greater degree of asymmetry with sharper onset or offset of the envelope, respectively. The direction of these asymmetries are mirrored for the upward and downward sweeps on all electrodes, and are consistent across all presentation levels with the clinical sound processor. However, the modulation rate and depth for each electrode was the same for upward and downward sweeps. The Discussion considers the extent to which the direction-dependent asymmetries may have provided a within-channel cue for the task.

**Fig. 2.**
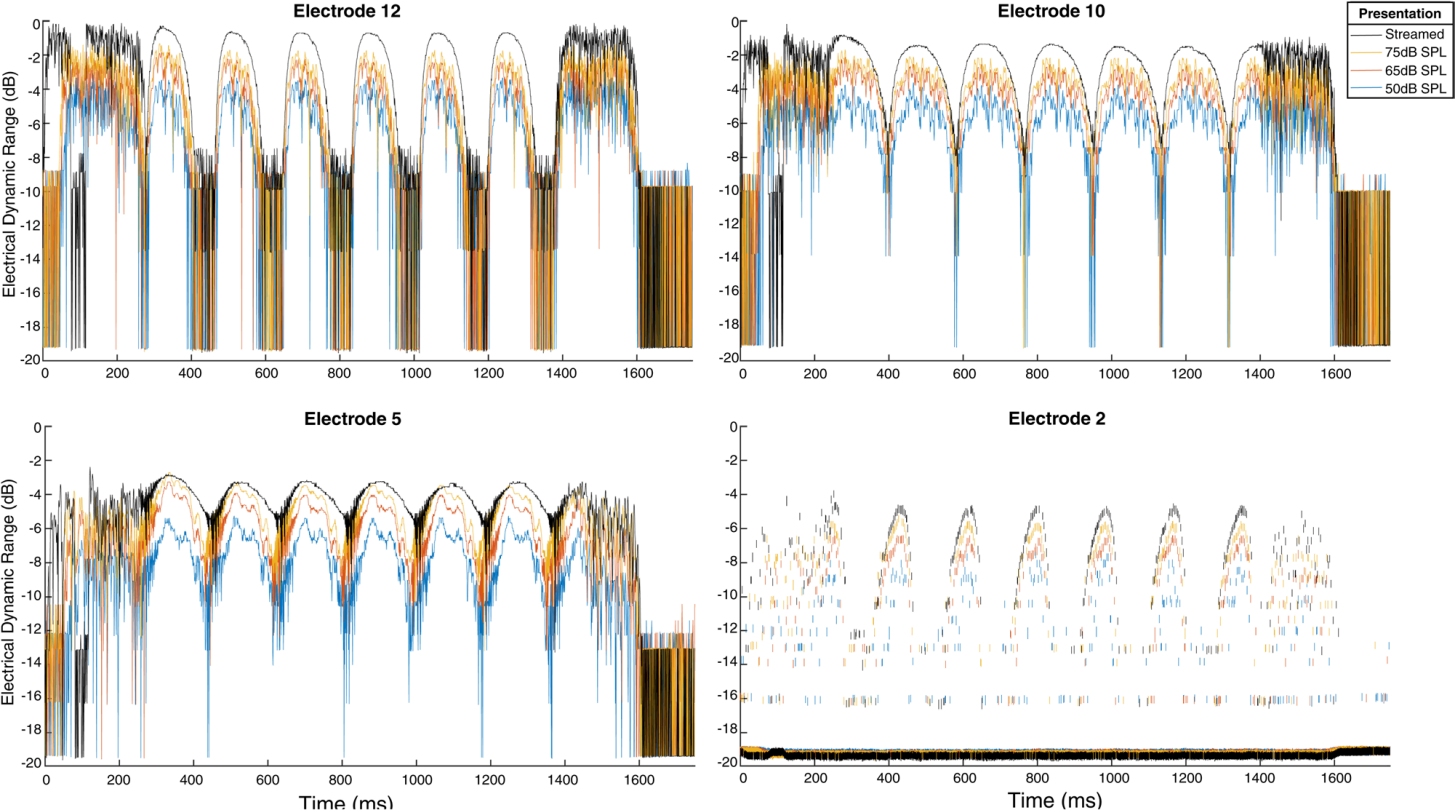
Voltage output expressed as the electrical dynamic range of the Med-El test map for a single downward stripe stimulus with a density of 5 on a single electrode. Each presentation condition is displayed as an individual line over time (msec), with the streamed stimulus in black, and for loudspeaker presentation at 75 dB SPL in yellow, at 65 dB SPL in red, and 50 dB SPL in blue. AGC and adaptive intelligence are enabled. AGC indicates automatic gain control.

Figure 2 depicts finer detail of the output from single electrodes (E12, E10, E5, and E2) in response to a downward sweeping STRIPES stimulus with a rippled density of 5, both for the streamed stimulus (shown in black) and for loudspeaker presentation at the three levels tested (75 dB SPL in yellow, 65 dB SPL in red, and 50 dB SPL in blue). The overall shape of the envelope in each channel is similar for the streamed and for all loudspeaker presentation levels, although the envelopes for the loudspeaker stimuli are less smooth and vary more from cycle to cycle. Although not shown here, disabling adaptive intelligence and AGC resulted in an overall lower output level when compared with having both enabled, and the output difference between each presentation level is substantially larger with both settings disabled.

### Effect of Presentation Level on STRIPES Density Thresholds

**Fig. 3.**
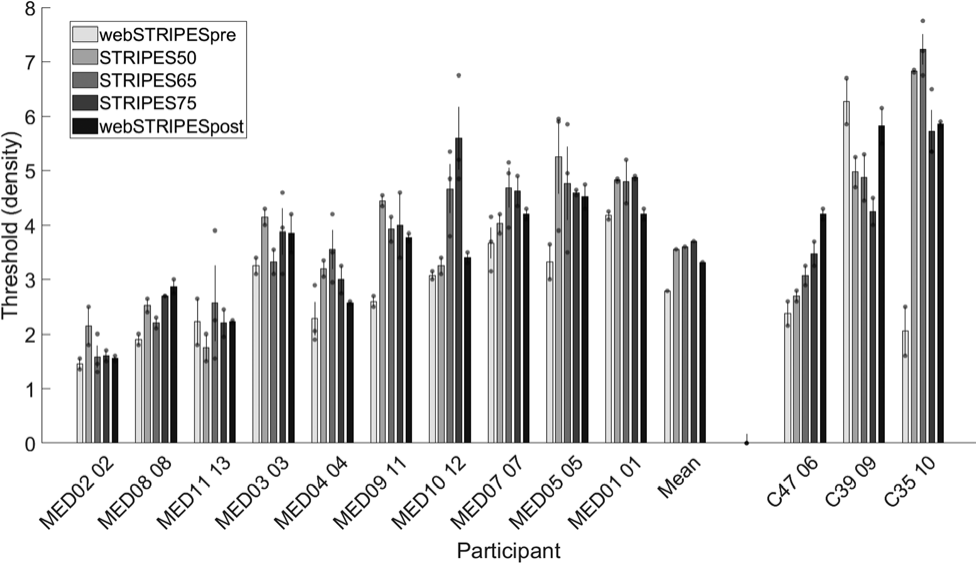
Each bar represents the mean ripple density threshold for the specified condition per participant. The markers indicate individual run thresholds with average error bars. The “mean” participant is the mean for all participants per test condition. Additional measures with Cochlear Ltd device recipients are displayed in the far right.

The mean RD threshold scores for each participant and condition are displayed in Figure 3. A repeated-measures ANOVA determined that mean RD thresholds were not significantly different between the presentation levels [*F*(2,18) = 0.236, *p* = 0.792] at the group level. Level effects were generally modest at the individual level: the mean difference in STRIPES threshold between the best and worst levels for each listener was −0.151 RD (95% confidence interval: −0.779 to −0.477), with only subject MED10 showing a relative difference exceeding 1 RD between any two levels. Unsurprisingly, stimulus level did affect loudness judgments (not shown), with median (inter-quartile range) perceived loudness levels for the 50, 65, and 75 dB SPL of 4 (3.75 to 5.0), 7 (6.0 to 7.25), and 8 (8 to 9), respectively, and as confirmed by a Friedman test [χ^2^(2) = 20.0, *p* < 0.001]. This pattern of results shows that while there was both a physical and perceptual difference in loudness for each presentation level, this had no significant effect on STRIPES density thresholds at a group level and usually modest effects at the individual level.

### Comparison of STRIPES and webSTRIPES

Before comparing STRIPES and webSTRIPES, a paired sample *t* test was used to check for any learning effect between the pretest and posttest webSTRIPES measurements. Participants had a poorer density threshold at pretest webSTRIPES (*M* = 2.7945 SD = 0.8473) compared with posttest webSTRIPES (*M* = 3.31750, SD = 0.9763) with a statistically significant difference of −0.523 (95% confidence interval: −0.850 to −0.195), *t*(9) = −3.614, *p* = 0.006, *d* = 0.457. Intraclass correlation coefficients using absolute-agreement, two-way mixed-effects model were completed using the individual trials from pretest (two runs) and posttest (two runs) webSTRIPES. In two instances of the pretest webSTRIPES, a third run was used which was excluded from the analysis. The test-retest reliability was calculated as an intraclass correlation coefficient of 0.929 with a 95% confidence interval of 0.789 to 0.981 (*p* < 0.001) indicating good-to-excellent reliability. Taken together, these results indicate that the webSTRIPES test was highly reliable albeit with a small learning effect that could not be explained by random variation of results within each participant.

**Fig. 4.**
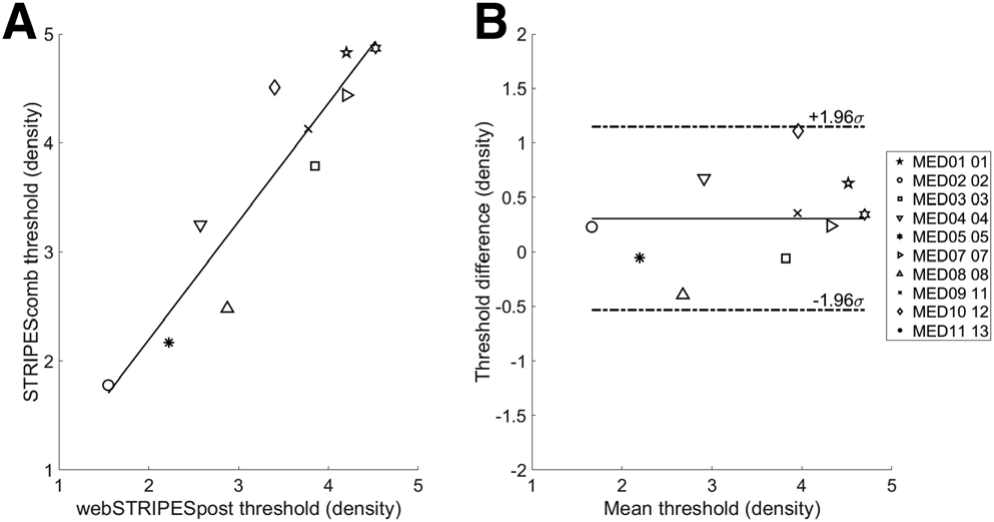
Correlation (A) and Bland–Altman (B) plot for mean thresholds calculated from combined STRIPES and posttest webSTRIPES. Solid and dashed lines in (B) show the mean and ±1.96 times the standard deviation of the threshold difference. STRIPES indicates Spectro-Temporal Ripple for Investigating Processor Effectiveness.

Given no significant differences were observed across presentation levels in the STRIPES loudspeaker measurements, the data were combined and averaged to assess the relationship with posttest webSTRIPES results. Figure 4 shows the correlation (A) and Bland–Altman plot (B) between the combined loudspeaker STRIPES thresholds and the posttest webSTRIPES results. The results are significantly correlated across participants (*r* = 0.894, df = 8, 95% confidence interval [0.84 to 0.93], *p* < 0.001), and the Bland–Altman plot shows a stable relationship between combined STRIPES and posttest webSTRIPES outcomes with consistent error across the different levels of performance [*F*(1,8) = 1.427, *p* = 0.266, *R*^2^ = 0.151]. All differences in threshold were inside the mean difference ±1.96 SD specified by Bland and Altman (1986) and corresponded to an RD difference less than or equal to about 0.7. Overall, this means that performance on webSTRIPES was comparable to loudspeaker STRIPES.

### Comparison of STRIPES and CRM Tests

**Fig. 5.**
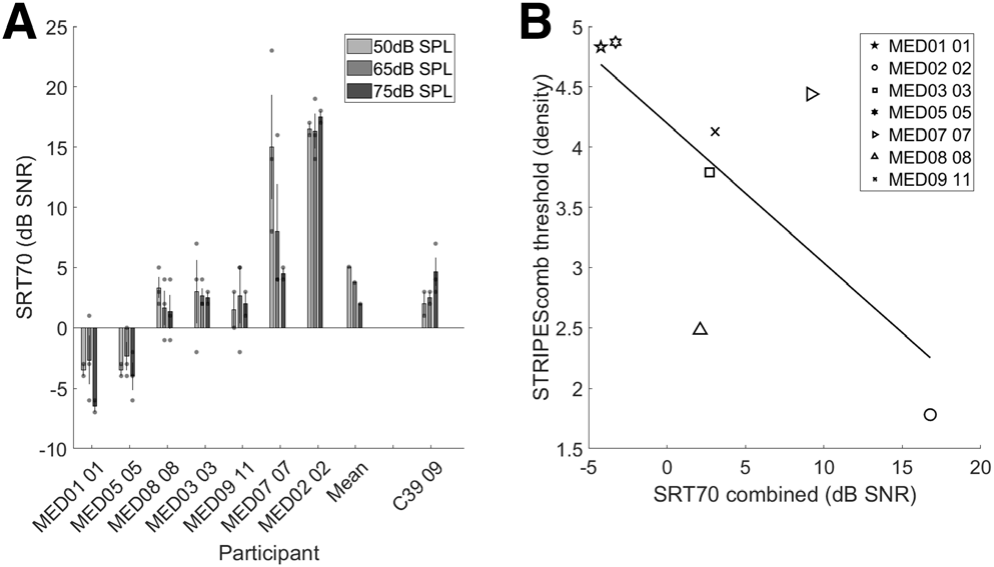
Individual SRT70 scores and SRT70/STRIPES correlation plot. A, Each bar represents the dB SNR for SRT70 for the specified condition per participant. The markers indicate individual run thresholds with average error bars. The “mean” participant is the mean for all participants per test condition (n = 7). B, Spearman’s rho correlation.

Seven Med-El participants returned for the second session and completed CRM testing (Fig. 5); three were unable to return for personal reasons. An RM-ANOVA was conducted to assess the effect of level on SRT70 and was not significant [*F*(2,12) = 1.135, *p* = 0.354]. This result remained nonsignificant even after removing MED02, whose SRT70 was very high (poor) at all presentation levels. An analysis of covariance was conducted to examine the relationship between participants’ CRM scores and their corresponding STRIPES scores across different levels. Variation in CRM score across level for a given participant could not be explained by the variation in their STRIPES score across level [*F*(1,13) = 2.274, *p* = 0.121]; for example, participant MED08 showed a large monotonic reduction in SRT70 with increasing level but only a modest and non-monotonic effect of level on their STRIPES density threshold. This lack of significant effect at an individual level is perhaps not surprising, given the generally very small/absent effects of level on the two tasks for most listeners. Finally, given no significant difference between presentation levels, individual-participant SRT70 results were averaged across presentation level, then compared with the combined STRIPES results (Fig. 5B). A nonparametric statistical analysis was completed using Spearman correlation (rho) as the data were not normally distributed and to avoid the results being dominated by one or more outliers. The combined CRM and STRIPES thresholds showed a monotonic correlation in the predicted direction, wherein good (high) STRIPES thresholds were related to good (low) SRT70s for the CRM test, but this was not statistically significant (rho = −0.607, n = 7, *p* = 0.148). This was likely due to the involvement of only 7 participants, leading to very wide 95% confidence intervals of −0.937 to 0.295.

### Additional Measures

As an additional measure, 3 participants who had received the Cochlear CI system were incorporated into the study (Fig. 3). This decision was motivated by difference in the behavior of the AGC and compression characteristics across the Cochlear and Med-El devices, alongside the peak picking patterns inherent to an N-of-M strategy. The results were broadly consistent with those of the Med-El device recipients, with the mean STRIPES condition outcomes being similar. The exception was the post-webSTRIPES condition, which showed a higher RD difference compared with the pre-webSTRIPES condition. This increase was largely driven by participant C35, who experienced nearly a 185% improvement. Including the Cochlear participants in the main analysis did not affect the statistical significance of the outcomes, except for the combined STRIPES and SRT70 correlation, where the inclusion of the only returning cochlear participant (C39) resulted in a significant correlation (rho = −0.738, n = 8, *p* = 0.037, 95% confidence interval: −0.952 to −0.044).

**Fig. 6.**
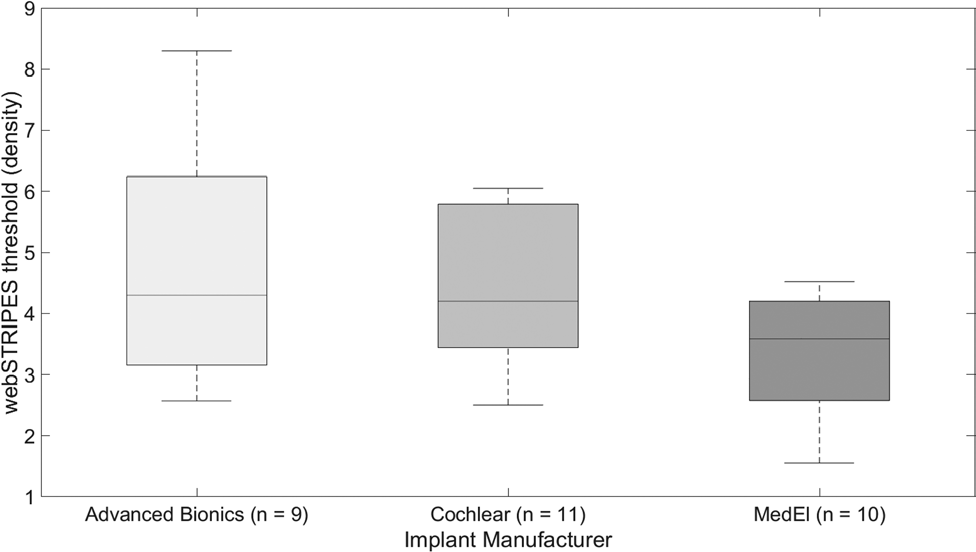
Box plot of mean webSTRIPES density thresholds by manufacturer. Data from Archer-Boyd et al. (2023) webSTRIPES results have been combined here. Participant numbers include Advanced Bionics (n = 9), Cochlear (n = 11), Med-El (n = 10).

Finally, using the webSTRIPES data from Archer-Boyd et al. (2023), we compare the mean webSTRIPES RD threshold results with the three device manufacturers (Advanced Bionics, Cochlear, and Med-El) (Fig. 6). The distribution of RD thresholds for the Med-El participants overlaps with that for the other devices [*F*(2,25) = 2.683, *p* = 0.088], albeit with a possible trend for the RD scores for the Med-El participants in the present study to be lower than those for Cochlear and AB participants reported by Archer-Boyd et al. (2023).

## DISCUSSION

### Effect of Presentation Level on STRIPES Performance

Our study extends investigations of the STRIPES test in CI listeners by examining level effects, as well as its use with a previously untested cochlear implant system. Despite participants ranking the presentation levels as perceptually different from one another, our findings support the notion that presentation level does not impact spectro-temporal processing ability when assessed by STRIPES in CI listeners. Previous studies of the effects of presentation level on nonspeech tests for spectral or spectro-temporal resolution in CI listeners have also generally failed to find a significant effect. de Jong et al. (2019) used the SMRT (Aronoff & Landsberger 2013) to assess the ability to detect changes in spectral RD of 11 AB implant recipients with a clinical and experimental strategy at two presentation levels of 45 and 65 dB. Although their analysis focused on differences between strategies, performance did not appear to differ between the two presentation levels tested (see Fig. 3 of de Jong et al. 2019). Won et al. (2011) performed an acoustic AM detection test and found no significant differences between thresholds at presentation levels at 50, 65, and 75 dB. Similarly, Brochier et al. (2017) assessed acoustic MDTs at 40, 50, and 60 dB in nine Cochlear CI system users and found no significant effect on level. This lack of a level-dependent effect is encouraging given that clinical applications of free-field testing are not as precise as research investigations, and that participants completing the webSTRIPES task adjust the presentation volume to varying levels.

### Comparison of STRIPES and webSTRIPES Performance

No significant difference was found in overall performance between the loudspeaker STRIPES and webSTRIPES thresholds for all 10 participants. Correlation analysis revealed a stable relationship between STRIPES and webSTRIPES outcomes, with consistent error across different levels of performance. In contrast to the findings of Archer-Boyd et al. (2023), in which 1 high-performing participant using the AB HiRes processing strategy may have exploited extraneous cues introduced by the Optima S strategy to surpass the spectral aliasing limit proposed by Winn & O’Brien (2022), the RD thresholds obtained for all participants in the present study remained below this estimated limit for both tests. In addition, both methods utilized the same number of reversals, suggesting equivalence between the approaches, potentially allowing for a shortened STRIPES task procedure.

The test-retest reliability of webSTRIPES was measured and revealed good-to-excellent reliability, consistent with previous reports of STRIPES reliability testing (Goehring et al. 2019; Archer-Boyd et al. 2020; van Groesen et al. 2023b). However, we observed an acute learning effect between pretest and posttest webSTRIPES assessments at the group level, albeit less than the variability observed in a repeat trial for the majority of listeners and consistent with previous reports of STRIPES (Goehring et al. 2019; Archer-Boyd et al. 2020; van Groesen et al. 2023b; van Groesen et al. 2023a). It seems plausible that a procedural learning effect is at play, given that participants had little-to-no experience with streamed stimuli, and there was no significant difference between post-webSTRIPES and all STRIPES conditions presented via loudspeaker. This has also been seen for the SMRT over a 6-week period with participants improving at 6 weeks compared with baseline (de Jong et al. 2018). Therefore, it might be beneficial for participants undertaking either STRIPES paradigm to undergo a full practice trial, which could then be excluded from the analysis, or to consider the total number of trials a participant completes and allow sufficient time between testing sessions to “wash out” any potential learning effects (Drennan et al. 2016). Our results, taken in context with previous findings, suggest either testing paradigm would enable comparable results, facilitating a straightforward assessment of spectro-temporal skills in individuals for clinical or novel research paradigms, thereby reducing travel or geographical barriers associated with repeated trips to a clinic or research facility (Bierbaum et al. 2020).

### Potential Use of Within-Channel Cues

As discussed in Electrodogram recordings and illustrated in Figure 2, the pattern of stimulation applied to the most basal and most-apical electrodes produced asymmetric envelopes, with the direction of the asymmetry differing between upward and downward stripes. Archer-Boyd et al. (2020) found that the AB Optima processing strategy, which uses current steering, and switching from direct input to loudspeaker presentation introduced asymmetries in the most-basal and most-apical channels. They reported that this did not produce a significant change in STRIPES density thresholds and concluded that, with the possible exception of 1 participant, it was unlikely that these asymmetries affected performance. We cannot be completely sure that a similar conclusion applies to our Med-El participants, partly because we did not include a control condition with no asymmetries, and partly because the asymmetry on electrode 2, which the FSP strategy stimulates using bursts of pulses at the zero-crossings, appears steeper than that observed by Archer-Boyd et al. (2020). Switching from streaming to loudspeaker presentation introduced more noise into the envelope, which might be expected to disrupt the use of cues based on envelope asymmetry, but which did not affect overall performance between either paradigm. If these asymmetries provided a sufficiently salient cue, we might have expected to see better performance for webSTRIPES compared with loudspeaker presentation. However, given the rate and depth of modulations were similar across channels and electrodes it seems unlikely that these asymmetries produce a salient enough cue to be reliably used. A possible explanation for the lack of performance differences in most listeners is that the spread of excitation is more variable and diffuse than the electrodograms suggest due to current spread within the cochlea, variations in electrode-to-modiolar distance, or other neural factors, which may have produced intersubject variations not detected within our sample.

### STRIPES and CRM Performance

There was a trend toward higher STRIPES density threshold scores related to lower SRT70 thresholds, but the correlation was not significant. The lack of significance in our study is likely due to the small sample size. Evidence in support of this interpretation comes from the large confidence interval associated with the correlation. Other studies have found significant correlations with speech-in-noise perception test and the STRIPES test including Goehring et al. (2019), who found significant correlations with speech-in-noise scores obtained with open-set stimuli (*R* = 0.59) and van Groesen et al. (2023b) who found a significant relationship with the Dutch matrix test (*R* = 0.53). In the same study, van Groesen et al. (2023b) observed a similar correlation between the Dutch matrix test and the SMRT test.

### Comparison of Device Manufacturer

This investigation assessed Med-El device users with the STRIPES stimuli in both loudspeaker and webSTRIPES format. Our results are consistent with the speculation put forth by Archer-Boyd et al. (2023) that the sparse nature of STRIPES stimuli would not lead to a marked difference between CIS and N-of-M strategies, which could be reflected by a significant difference between manufacturers. The Med-El and Cochlear data presented here are similar to previously published mean webSTRIPES density thresholds obtained from other device manufacturers. Equally, the introduction of FSP information and/or the extended low-frequency allocation of the filter bins in our Med-El participants does not appear to have resulted in STRIPES thresholds that differ markedly from other devices. Of course, these comparisons are made between different participants and it is possible that the different factors each had an effect but canceled each other out, so we cannot conclude that the strategy, array type, or preprocessing had no effect at all. Finally, we note that STRIPES density thresholds were obtained for all Med-El participants remained below the estimated limit of spectral aliasing as proposed by Winn and O’Brien (2022).

### Future Investigations

The understanding of how CI recipients learn to process the novel form of auditory stimulation provided by their device remains incomplete, particularly regarding the longitudinal development of spectro-temporal skills and their relationship with speech perception. Currently, the literature lacks a definitive nonspeech measure to track this maturation process over time among CI users (Drennan et al. 2016), with most assessing spectral or temporal skills despite work showing that CI users have better spectro-temporal sensitivity than can be predicted by spectral-only and temporal-only sensitivity (Zheng et al. 2017). Utilizing STRIPES as a tool could offer valuable insights into the various contributions of underlying mechanisms driving auditory-based speech perception implicated in learning how to use electrical stimulation, providing a deeper comprehension of the timeline for optimizing CI functionality, and subsequently optimizing novel stimulation strategies to optimize these cues. In addition, assessments using STRIPES have all been conducted with postlingually deafened adult CI recipients and there may be differences between prelingually deafened adult recipients (Boisvert et al. 2020), as well as pediatric recipients during their auditory system maturation (Gifford et al. 2018). Indeed, one of the largest studies to date investigating spectral and temporal skills in pediatric CI recipients aged 4 to 13 (mean age = 8.33) years old, found neither spectral or temporal resolution were significantly correlated with speech recognition (DeFreese et al. 2024). However, they did not include a combined spectro-temporal task and considered each separately. They also found that spectral resolution significantly increased with age and CI experience. The STRIPES test could be a suitable solution in clinical and research settings for examining a potential contribution of these cues across these groups. An open question also remains to the benefit of fine temporal processing as employed by the FSP strategy on the STRIPES test, or indeed on other measures of spectral and/or temporal skills. Further work could investigate the influence of FSP while holding the frequency allocation constant, or indeed in comparison of new anatomy-based fitting strategies (Creff et al. 2024; Walia et al. 2024), which might reduce place pitch stimulation error.

## CONCLUSIONS

The effect of presentation level on the STRIPES test was investigated with Med-El device users, and performance compared with the webSTRIPES version. Consistent with other tests of spectral modulation detection, no significant effect of presentation level was found in CI listeners at the group level. WebSTRIPES had good-to-excellent test-retest reliability and a high level of agreement between either test paradigm when using the Med-El CI system. However, variations in the presentation level did not explain why some people did better or worse in the speech-in-noise test at various levels. Further, webSTRIPES results appear broadly similar between device manufacturers, positioning the test as a potential language-independent tool for comparing outcomes for CI listeners’ spectro-temporal processing regardless of device manufacturer.

## ACKNOWLEDGMENTS

This work was supported by the Medical Research Council Grant No. G116768 to R.P.C. The funding organization had no role in the design and conduct of the study, in the collection, analysis, and interpretation of the data, or in the decision to submit the article for publication, or in the preparation, review, or approval of the article. Information on how to download and use the STRIPES test can be obtained from https://www.hearing-research.group.cam.ac.uk/software/.
